# Ailanthus

**Published:** 1887-12

**Authors:** A. McNeil

**Affiliations:** San Francisco


### AILANTHUS.

A. McNeil, M. D., San Francisco.

Ailanthus is one of the new remedies, but, unlike many others, it has been well proven. It deserves more clinical use than is given to it; hitherto its field of usefulness has been confined almost exclusively to scarlatina. Its provings show it is also adapted to diphtheria, to typhoid conditions, to organic diseases of the heart, and the like, when properly indicated.

The type of scarlatina to which this drug is adapted is almost the opposite of that in which Bell. is useful. In the latter every function is morbidly active, for instance, the delirium, the circulation, etc. With Bell. the mucous membranes, the skin, and eruption are bright red from active arterial congestion. The skin when pressed upon by the finger is pale, but the redness returns immediately the pressure is removed. With Ailanthus this is reversed; the parts are purple from sluggish venous congestion, and the circulation returns very slowly when skin is pressed upon. (And yet some who claim to be scientific homœ-

opaths would give Bell. to all cases of scarlatina, and claim Hahnemann as their authority. A study of Bell. in his *Materia Medica Pura* will readily refute this assertion.) With Bell. the eruption is smooth and the skin bright red and *hot* to touch; with Ailanthus, the eruption is rough, of purple color, in patches, with purplish skin between and sudden prostration. With Bell., the delirium is active, patient wants to bite, strike, or tear; with Ailanthus the patient is semi-conscious, does not comprehend what is said; or there is stupor, delirium, and insensibility; muttering delirium with sleeplessness. The throat is livid, swollen; the tonsils are prominent and studded with deep ulcers, oozing a scanty fetid discharge. External neck swollen and sensitive. The tongue is livid on the tips and edges. The countenance indicates much distress and there is great prostration. Stools and urine pass involuntarily (like Arn. and Hyos.).

The eruption is miliary, dark, and sometimes interspersed with vesicles; it may be slow in appearing and scanty.

In diphtheria, Ailanthus is indicated when the throat is purple, tender, and sore (Lach.), on inhaling air; external throat swollen and sensitive (Lach.) and accompanied by great prostration.

Typhoid states call for Ailanthus when the skin, the tips and edges of the tongue are purple, combined with the mental states already noticed; also hot face, dizziness, cannot sit up, as it produces vertigo, nausea, and vomiting (like Bry.).

Ailanthus has no clinical history in the treatment of organic diseases of the heart; yet its characteristics show its adaptability to certain forms with purple skin, dull pain, and contracted feeling about base of heart and through centre of left lung; also weak, irregular pulse, numbness of left arm. (Aconite has this and Rhus aching in it.) Tingling of arms and fingers (Acon.) on waking.

Attention has been called to only a part of the Ailanth. symptoms; it covers many cases when its peculiar symptoms are found.

